# Growth differentiation factor 15 mediates epithelial mesenchymal transition and invasion of breast cancers through IGF-1R-FoxM1 signaling

**DOI:** 10.18632/oncotarget.21765

**Published:** 2017-10-10

**Authors:** Bridgette F. Peake, Siobhan M. Eze, Lily Yang, Robert C. Castellino, Rita Nahta

**Affiliations:** ^1^ Molecular & Systems Pharmacology PhD Program, Graduate Division of Biological and Biomedical Sciences, Emory University, Atlanta, GA, USA; ^2^ Department of Pharmacology, School of Medicine, Emory University, Atlanta, GA, USA; ^3^ Department of Pediatrics, School of Medicine, Emory University, Aflac Cancer & Blood Disorders Center, Children’s Healthcare of Atlanta, Atlanta, GA, USA; ^4^ Department of Hematology & Medical Oncology, School of Medicine, Emory University, Atlanta, GA, USA; ^5^ Department of Surgery, School of Medicine, Emory University, Atlanta, GA, USA; ^6^ Winship Cancer Institute, Emory University, Atlanta, GA, USA

**Keywords:** breast cancer, signaling, invasion

## Abstract

Expression of the inflammatory cytokine growth differentiation factor 15 (GDF15) is significantly elevated in many tumor types in association with epithelial mesenchymal transition (EMT), drug resistance, and progressive disease. However, few studies have examined GDF15 expression, signaling, or function in breast cancer. In the current study, we demonstrate that GDF15 is associated with high tumor grade, ER-negativity, and HER2 overexpression in patients with breast cancer. Stable overexpression of GDF15 upregulates expression of mesenchymal markers and transcription factors, including FoxM1, and increases cellular invasion. GDF15 stable clones and breast cancer cells stimulated with recombinant human GDF15 (rhGDF15) demonstrate activation of insulin-like growth factor-1 receptor (IGF-1R), EMT, and invasion. Pharmacologic inhibition of IGF-1R reduces GDF15-mediated EMT and invasion in stable clones, and FoxM1 knockdown rescues invasion and EMT in GDF15 stable clones and rhGDF15-stimulated cells. These data suggest that IGF-1R-FoxM1 signaling is a potential mechanism through which GDF15 drives EMT and invasion of breast cancers. Further, GDF15 knockdown significantly inhibits invasion of HER2-overexpressing and triple-negative breast cancer cells, supporting further preclinical investigation of GDF15-targeted therapies.

## INTRODUCTION

Growth differentiation factor 15 (GDF15; also referred to as macrophage inhibitory cytokine-1, MIC-1, and nonsteroidal anti-inflammatory drug activated gene-1, NAG-1) is a stress-induced inflammatory cytokine [1, 2]. Under normal physiological conditions, GDF15 is expressed abundantly only in the placenta. However, stressors, such as inflammation and injury, induce GDF15 expression in epithelial cells, fibroblasts, and macrophages. In addition, many pathologic conditions, including insulin resistance, diabetes, cardiovascular disease, impaired cognitive ability, and malignancies, are associated with elevated levels of circulating GDF15 [3–5]. Patients with advanced-stage cancers, including breast, express high levels of GDF15 in tumor tissues and/or sera [6–10].

GDF15 shares structural features with members of the transforming growth factor-beta (TGF-beta) superfamily [1], and was recently demonstrated to bind with high affinity to the orphan receptor glial-derived neurotrophic factor-family receptor-alpha-like (GFRAL) [11–13]. GDF15 overexpression stimulates PI3K/Akt/mTOR and MEK/ERK signaling [14–17], and promotes epithelial to mesenchymal transition (EMT) in colorectal and ovarian cancer cells [18, 19]. GDF15 promotes the acquisition of cancer stem cell-like properties in breast cancers [16], and confers resistance to HER2-targeted therapy in breast cancer [14]. In the current study, we demonstrate that GDF15 promotes EMT and invasiveness of breast cancers through insulin-like growth factor-1 receptor (IGF-1R) signaling and transcription factor FoxM1 upregulation.

## RESULTS

### GDF15 expression correlates with ER-negative and HER2-positive status in patients with breast cancer

**Figure 1:**
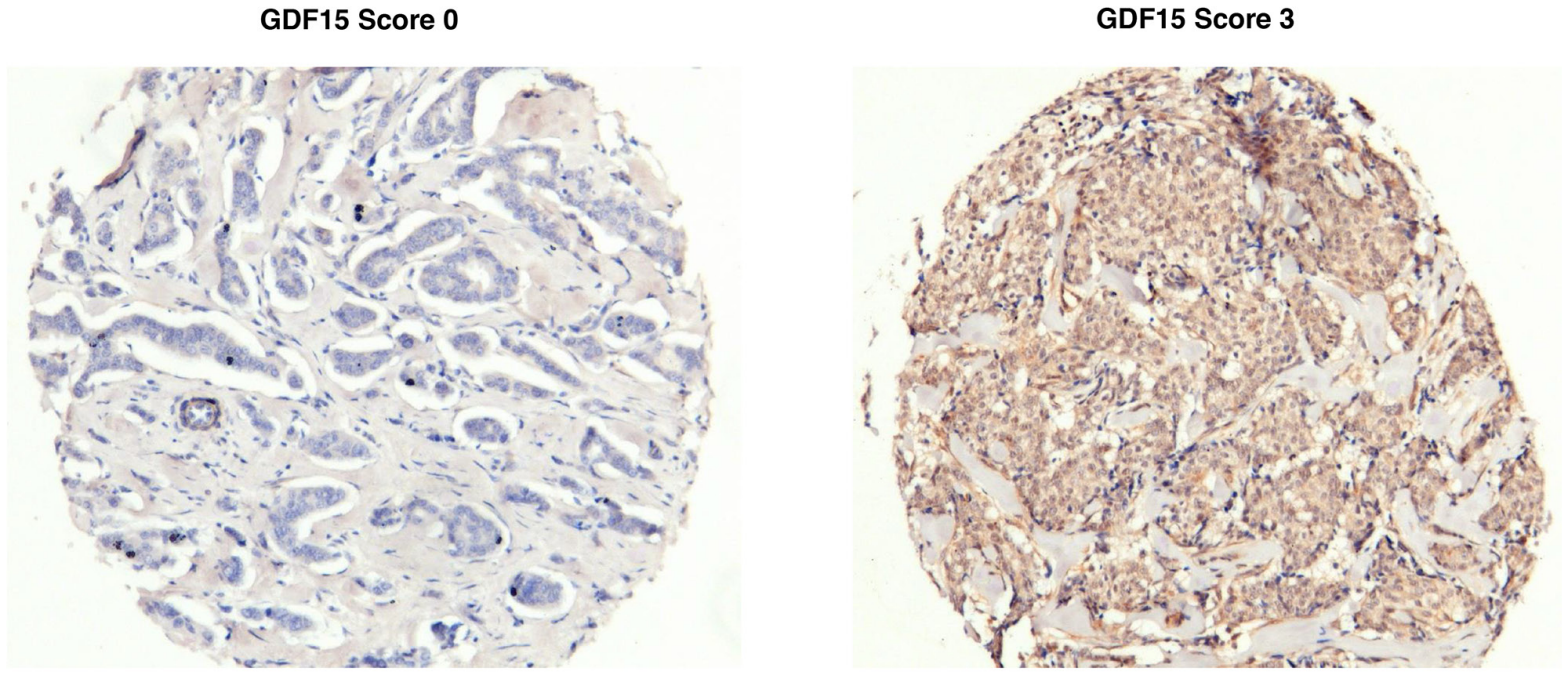
Representative GDF15 immunohistochemical (IHC) staining Representative images of breast tumor tissues scored as 0 (*left*) or 3 (*right*) for GDF15 IHC staining.

GDF15 expression, as assessed by IHC (Figure 1), was correlated with baseline clinical characteristics in a cohort (N=592) of patients with breast cancer (Table 1). The majority of patients were older than 50 years (75%) and had low-grade tumors (68%). GDF15 expression (staining score ≥ 1) was observed in approximately two-thirds (65%) of patients. GDF15-positive scoring correlated with high tumor grade (P=0.002), as approximately 75% of patients with high-grade tumors displayed a GDF15 score of 1+. Most (87%) patients in the cohort had fewer than four lymph node metastases. Stratification of patients based on ≤ 3 lymph node metastases versus > 3 lymph node metastases did not show significant correlation with GDF15 staining. The molecular subtypes of breast cancers represented in the cohort were consistent with published literature [6, 20], with 68% estrogen receptor (ER)-positive tumors and 13% human epidermal growth factor receptor 2 (HER2)-positive. A majority of patients in each subtype expressed GDF15, with a statistically higher percentage of ER-negative and HER2-positive tumors exhibiting GDF15-positivity (P=0.03 for each).

**Table 1:** Correlations between GDF15 IHC score and clinical characteristics in patients with breast cancer

Characteristic, n (%)	N=592	GDF15 IHC score		P value
		0 (n=204)	1+ (n=388)	
Age (years)				0.7
< 50	147 (24.8)	48 (32.7)	99 (67.3)	
≥ 50	445 (75.2)	156 (35.1)	289 (64.9)	
Grade				**0.002**
1-2	402 (67.9)	156 (38.8)	246 (61.2)	
3	190 (32.1)	48 (25.3)	142 (74.7)	
Lymph node (#)				0.3
≤ 3	515 (87.0)	173 (33.6)	342 (66.4)	
> 3	77 (13.0)	31 (40.3)	46 (59.7)	
ER				**0.03**
Negative	192 (32.4)	54 (28.1)	138 (71.9)	
Positive	400 (67.6)	150 (37.5)	250 (62.5)	
HER2				**0.03**
Negative	517 (87.3)	187 (36.2)	330 (63.8)	
Positive	75 (12.7)	17 (22.7)	58 (77.3)	

Abbreviations: ER, estrogen receptor; GDF15, growth differentiation factor 15; HER2, human epidermal growth factor receptor 2; IHC, immunohistochemistry

### GDF15 overexpression alters cell cycle profiles and induces EMT in breast cancer

**Figure 2:**
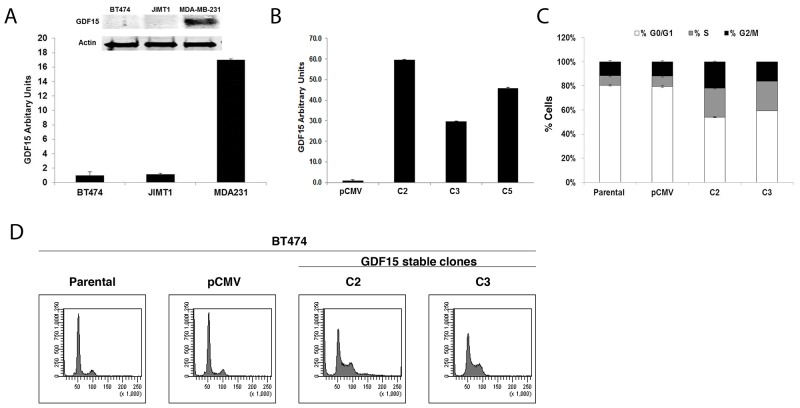
GDF15 overexpression alters breast cancer cell cycle profile **(A)** Western blotting (*above*) and real-time PCR (*graph*) for GDF15 in BT474, JIMT1 and MDA-MB-231 (MDA231) breast cancer cell lines. PCR values reflect fold change in *GDF15* transcript normalized to *RPLPO* housekeeping gene. Error bars represent standard deviation between triplicate samples; experiments were repeated at least 3 times. **(B)** Real-time PCR for *GDF15* in BT474 pCMV stable empty vector control clone (pCMV) and GDF15 stable clones 2, 3, and 5 (C2, C3, and C5). Values reflect fold change in *GDF15* transcript level normalized to *RPLPO* housekeeping gene. Error bars represent standard deviation between triplicate samples; experiments were repeated at least 3 times. **(C-D)** BT474 parental, pCMV empty vector control clone (pCMV), and GDF15 stable clones 2 and 3 (C2, C3) were fixed, stained with propidium iodide, and analyzed for DNA content by flow cytometry. The percentage of cells in each cell cycle phase is shown per cell line (C) (white, G0/G1; gray, S; black, G2/M). Error bars represent standard deviation between triplicate samples; experiments were repeated at least 3 times. Representative cell cycle histograms are shown per line (D).

BT474 and JIMT1 breast cancer cells express low levels of GDF15, whereas MDA-MB-231 (MDA231) breast cancer cells demonstrate high endogenous expression of GDF15 (Figure 2A). Stable overexpression of GDF15 in BT474 (Figure 2B) resulted in a 3-fold increase in the percentage of cells in S phase compared with parental and empty vector control cells (Figure 2C–2D).

**Figure 3:**
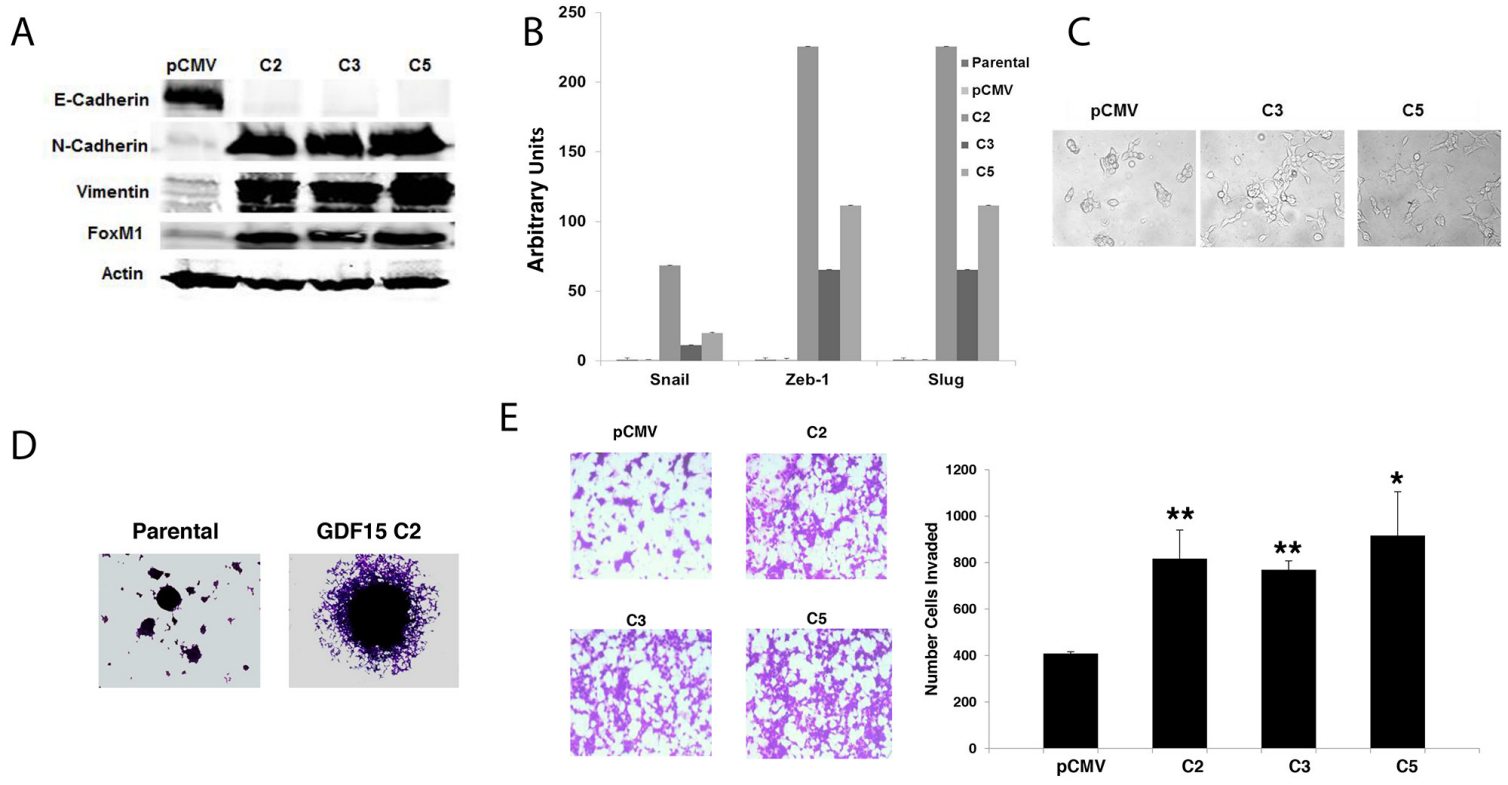
GDF15 induces epithelial mesenchymal transition and invasion in breast cancer cells **(A)** Total protein whole-cell lysates were collected from BT474 pCMV stable empty vector control clone (pCMV) and GDF15 stable clones 2, 3, and 5 (C2, C3, and C5). Western blots were performed for E-Cadherin, N-Cadherin, vimentin, and FoxM1; actin was measured as a loading control. Blots were repeated at least three times, and representative blots are shown. **(B)** Real-time PCR for Snail, Zeb-1 and Slug in BT474 parental, pCMV stable empty vector control clone (pCMV) and GDF15 stable clones 2, 3, and 5 (C2, C3, and C5). Values reflect fold change in transcript normalized to *RPLPO* housekeeping gene. Error bars represent standard deviation between triplicate samples; experiments were repeated at least 3 times. **(C)** BT474 pCMV empty vector control clone (pCMV) and GDF15 stable clones 3 and 5 (C3 and C5) were imaged at 10× magnification to evaluate changes in morphology. **(D)** Representative images of spheroid cultures are shown for BT474 parental and GDF15 stable clone 2 (C2). **(E)** BT474 stable empty vector control clone (pCMV) and GDF15 stable clones 2, 3, and 5 (C2, C3, and C5) were plated in basement membrane matrix mimic (Matrigel)-coated Boyden chambers in serum-free media; 10% FBS was added to the well below each chamber as a chemo-attractant. After 24 hours, cells were fixed and stained. Representative photos of invading cells are shown at 20× magnification. The total number of invading cells was counted in 10 random fields; the average number of invading cells is shown for triplicate cultures per cell line; student’s t-test, ^**^p<0.005, ^*^p<0.05.

We previously found that GDF15 induces EMT in ovarian cancer cells [18]. To determine if stable GDF15 overexpression also promotes EMT in breast cancer cells, we examined expression of mesenchymal and epithelial markers by western blotting. Stable GDF15-overexpressing clones exhibited dramatic downregulation of the epithelial marker E-cadherin, and upregulation of mesenchymal markers N-cadherin, vimentin, and transcription factor FoxM1 (Figure 3A). Expression levels of mesenchymal transcription factors Snail, Zeb-1, and Slug were also increased (Figure 3B), with phenotypic morphological changes consistent with acquisition of a mesenchymal phenotype (Figure 3C). EMT is associated with increased potential for migration and invasion. Consistent with this concept, stable overexpression of GDF15 conferred spheroid-forming capability to breast cancer cells, in contrast to parental cells, which did not form spheroids in 3-d culture (Figure 3D). GDF15-overexpressing breast cancer cells also demonstrated significantly increased invasion through basement membrane matrix (Figure 3E).

### IGF-1R-FoxM1-MMP signaling underlies GDF15-mediated EMT in breast cancer

**Figure 4:**
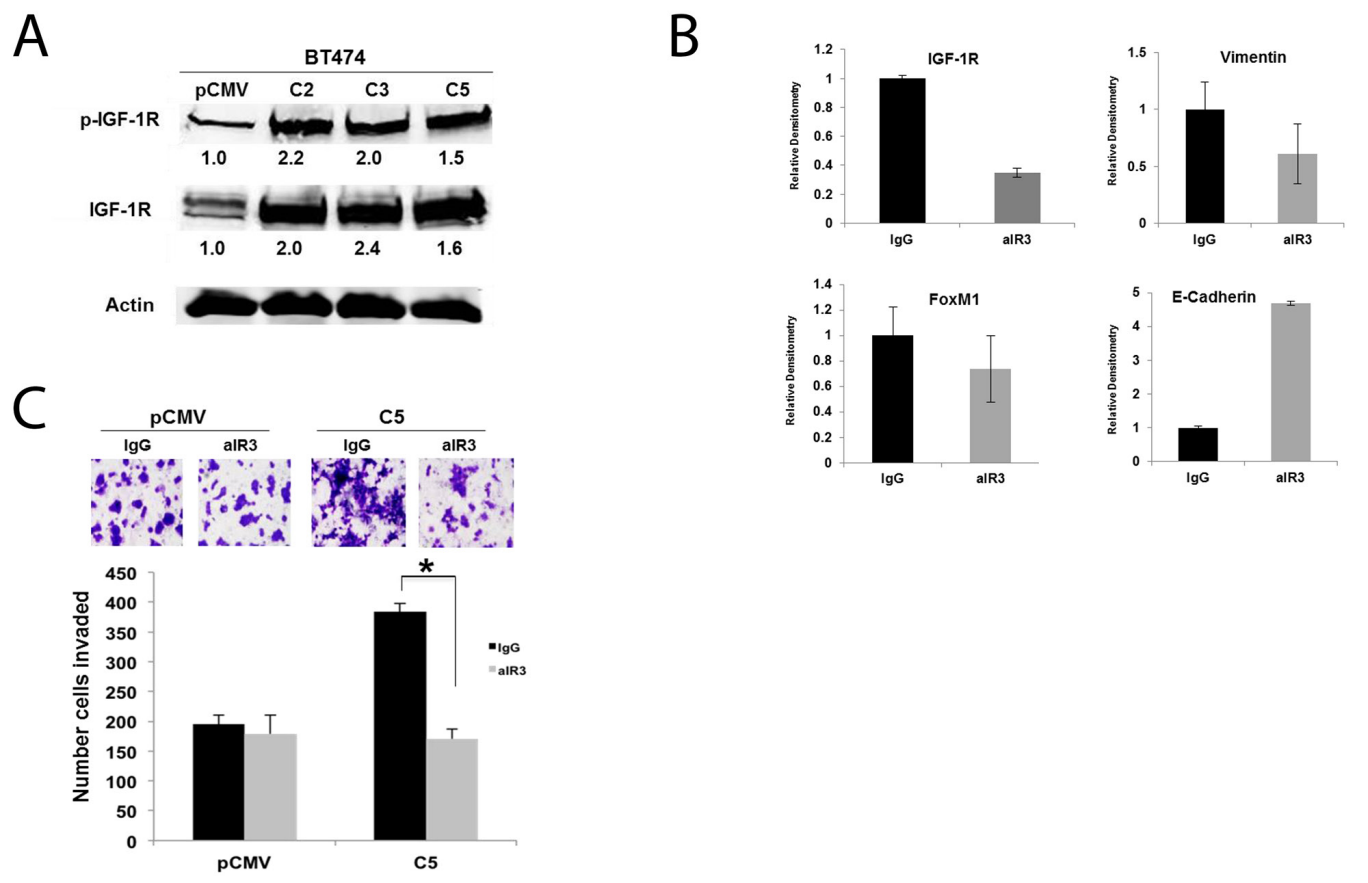
IGF-1R activation contributes to EMT and invasion of GDF15-overexpressing breast cancer cells **(A)** Total protein lysates were collected from BT474 stable empty vector control clone (pCMV) and GDF15 stable clones 2, 3, and 5 (C2, C3, and C5). Western blots were performed for p-Tyr1131 IGF-1R and total IGF-1R; actin was probed as loading control. Experiments were repeated 3 times; representative blots are shown. Quantification (shown beneath each band) was normalized to actin and performed using Odyssey Li-Cor imaging software. **(B)** BT474 GDF15 stable clone 2 (C2) cells were treated with normal mouse IgG control or 0.25 μg/mL alpha IR3 (aIR3) IGF-1R monoclonal antibody for 48 hours. Western blots of total protein lysates were performed for total IGF-1R, vimentin, FoxM1, and E-Cadherin. Bar graphs show quantification relative to actin loading control, and was performed using Odyssey Li-Cor imaging software. Error bars represent standard deviation between triplicates; experiments were performed at least 3 times. **(C)** BT474 stable empty vector control clone (pCMV) and BT474 GDF15 stable clone 5 (C5) cells were pre-treated with normal mouse IgG or 0.25 μg/mL alpha IR3 (aIR3) IGF-1R monoclonal antibody for 24 hours. Cells were then seeded in Matrigel-coated Boyden chambers in serum-free media plus control IgG or aIR3; 10% FBS was added to the well as a chemo-attractant. After 24 hours of invasion, cells in chambers were fixed and stained. Representative photos of invading cells are shown at 20× magnification. The total number of invading cells was counted in 10 random fields; the average number of invading cells is shown for triplicate cultures per cell line, ^*^p<0.05.

We previously demonstrated the importance of IGF-1R as a major upstream mediator of breast cancer cell EMT and invasion [21, 22]. Stable overexpression of GDF15 resulted in ∼2-fold increase in total and phosphorylated IGF-1R relative to empty vector control (Figure 4A). Treatment of stable GDF15-overexpressing cells with IGF-1R-targeted antibody alpha IR3 reduced expression of IGF-1R and induced expression of epithelial marker E-cadherin (Figure 4B). IGF-1R inhibition also rescued the invasive phenotype of GDF15-overexpressing stable cells without significantly affecting control cells (Figure 4C). These results suggest that IGF-1R activation contributes to the EMT and invasiveness of GDF15-positive breast cancers.

**Figure 5:**
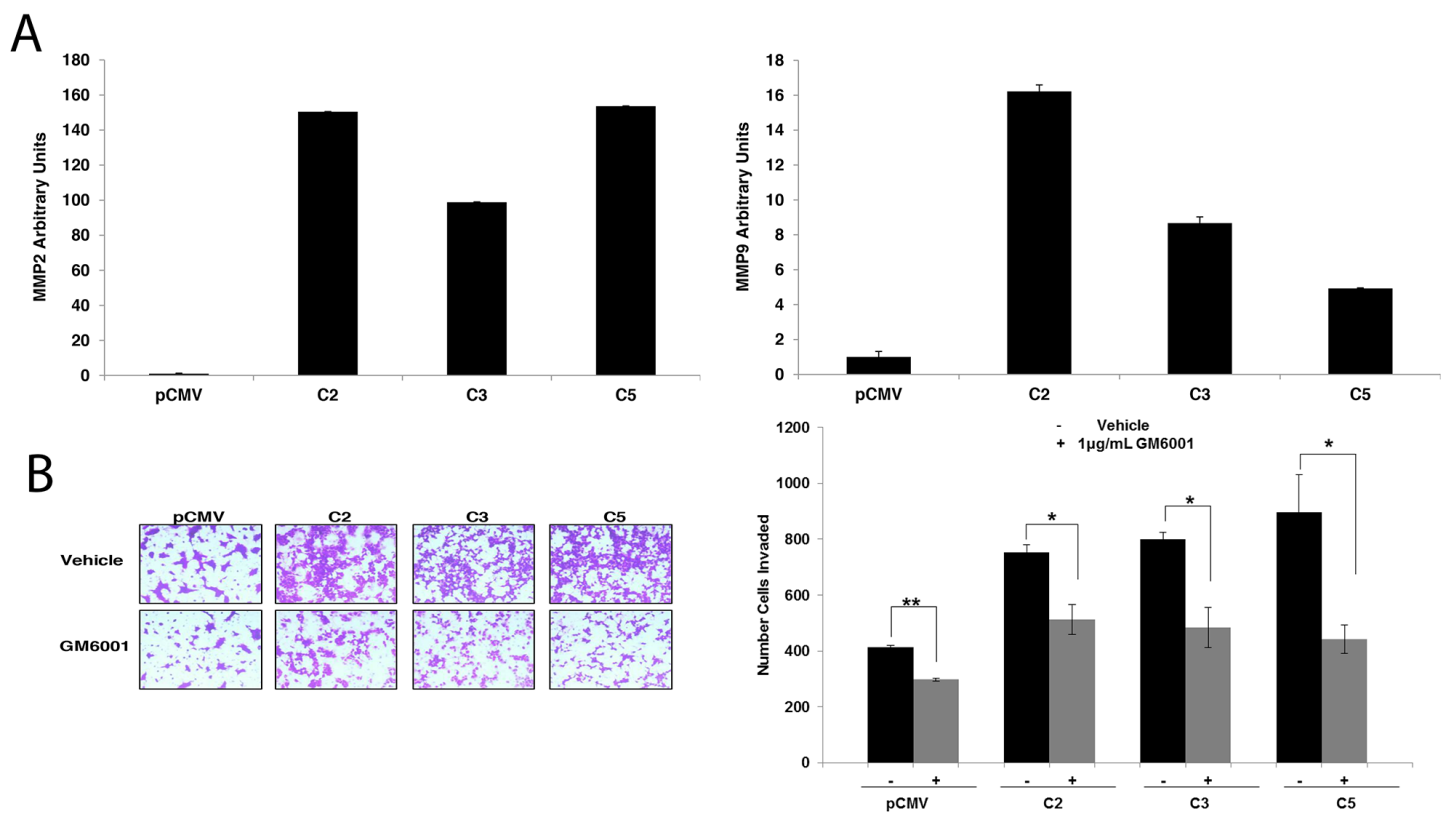
Matrix metalloproteinases (MMPs) mediate invasion of GDF15-overexpressing breast cancer cells **(A)** Real-time PCR of total RNA from BT474 pCMV empty vector control clone (pCMV) and GDF15 stable clones (C2, C3, and C5) for MMP2 (*left graph*) and MMP9 (*right graph*). Values reflect average fold in transcript normalized to internal control RPLPO relative to pCMV group. Error bars represent standard deviation between triplicate samples; experiments were repeated 3 times. **(B)** BT474 pCMV empty vector control clone (pCMV) and GDF15 stable clones (C2, C3 and C5) were plated in serum-free media in Matrigel-coated Boyden chambers and treated with vehicle control or 1 μg pan-MMP inhibitor GM6001 for 24 hours, after which cells were fixed and stained. Representative photos of invading cells are shown at 20× magnification. The total number of invading cells was counted in 10 random fields; the average number of invading cells is shown for triplicate cultures per cell line; student’s t-test, ^**^p<0.005, ^*^p<0.05.

To further elucidate the molecular mechanism through which GDF15 enhances breast cancer cell invasion, we measured expression levels of matrix metalloproteinases (MMP) MMP2 and MMP9, which are transcriptional targets of FoxM1 and mediators of cancer cell invasion. Stable GDF15-overexpressing clones demonstrated significant upregulation of MMP2 and MMP9 (Figure 5A). Treatment with the broad-spectrum MMP inhibitor GM6001 significantly reduced invasiveness of stable GDF15-overexpressing clones (Figure 5B), suggesting that MMPs contribute to the invasive phenotype of GDF15-overexpressing breast cancer cells.

**Figure 6:**
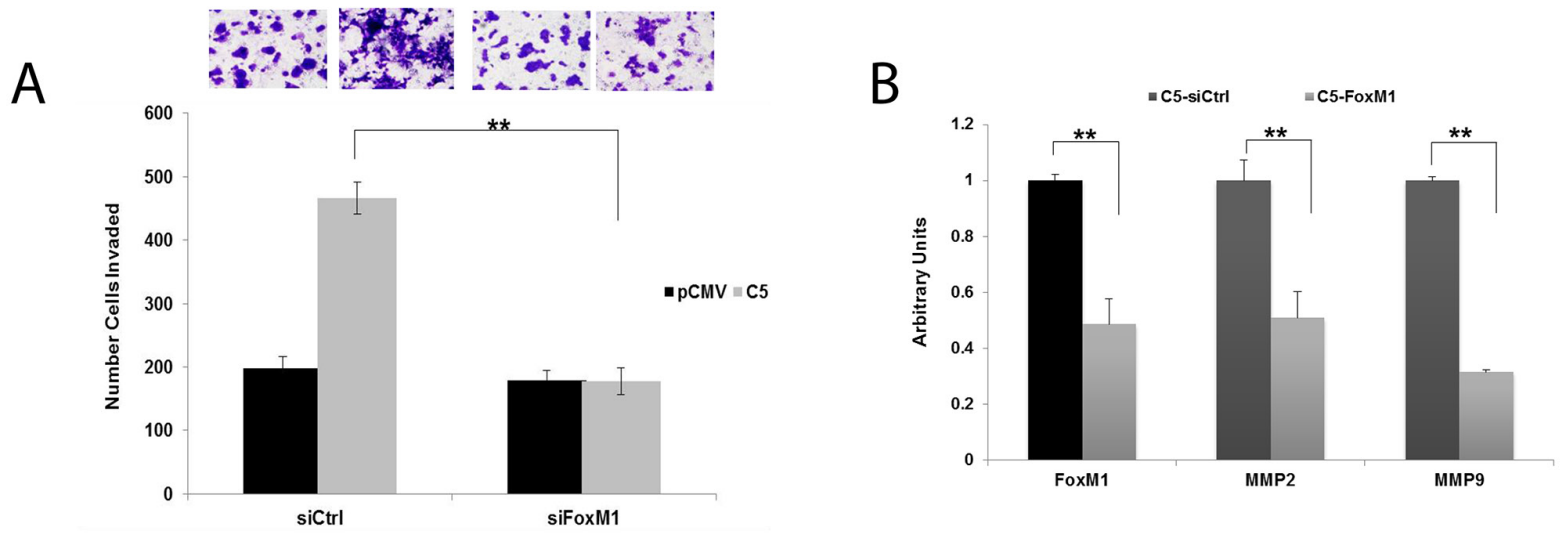
FoxM1 promotes invasion and upregulation of MMP2 and MMP9 in GDF15-overexpressing breast cancer cells **(A)** BT474 stable empty vector control clone (pCMV) and GDF15 stable clone 5 (C5) were transfected with 100 nM control siRNA (siCtrl) or FoxM1 siRNA (siFoxM1) for 48 hours, and then plated in serum-free media in Matrigel-coated Boyden chambers. After 24 hours, cells were fixed and stained. Representative photos of invading cells are shown at 20× magnification. The total number of invading cells was counted in 10 random fields; the average number of invading cells is shown for triplicate cultures per cell line; student’s t-test, ^**^p<0.005. **(B)** BT474 GDF15 stable clone 5 (C5) cells were transfected with 100 nM control siRNA (siCtrl) or FoxM1 siRNA (FoxM1) for 48 hours. Real-time PCR was performed for FoxM1, MMP2, and MMP9. Values reflect average fold in transcript normalized to internal control RPLPO. Error bars represent standard deviation between triplicate samples; experiments were repeated 3 times, ^**^p<0.005.

Increased FoxM1 expression contributes to cancer cell invasiveness [23]. To determine if FoxM1 upregulation contributes to the increased invasiveness observed in our model system, we transiently knocked down FoxM1 expression. Downregulation of FoxM1 significantly decreased invasion (Figure 6A) to the level of control cells, and reduced expression of MMP2 and MMP9 (Figure 6B).

**Figure 7:**
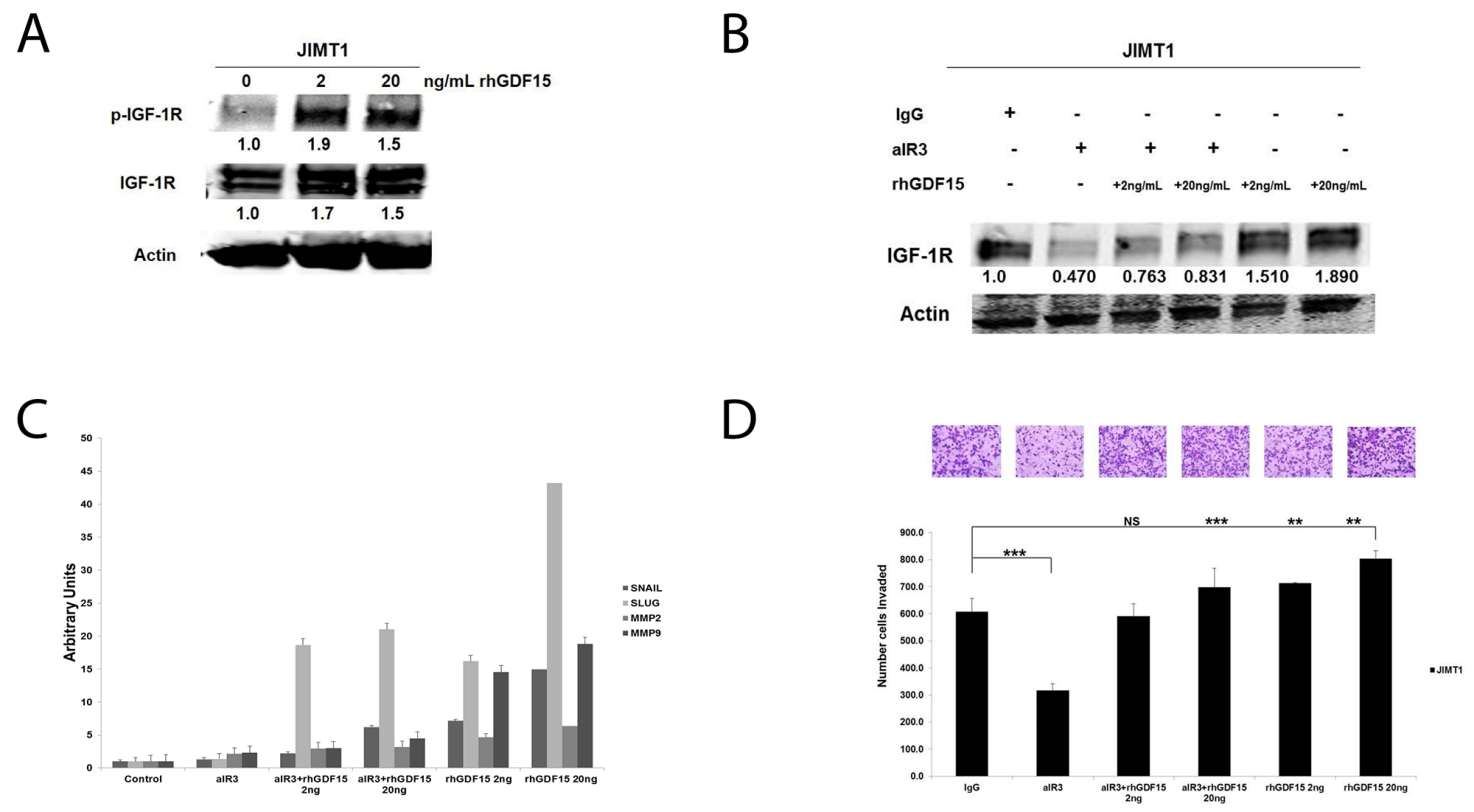
Effects of IGF-1R inhibition on GDF15-stimulated EMT and invasion **(A)** JIMT1 cells were serum starved for 24 hours, and then stimulated with 2 or 20 ng/mL of recombinant human GDF15 (rhGDF15) for another 24 hours. Western blots of total protein lysates are shown for p-Tyr1131 IGF-1R and total IGF-1R; actin was probed as loading control. Experiments were repeated 3 times; representative blots are shown. Quantification (shown beneath each band) was normalized to actin and performed using Odyssey Li-Cor imaging software. **(B)** JIMT-1 cells were pretreated for 24 hours with control IgG or 0.25 μg/ml alpha IR3 (aIR3) in serum-free media, and then stimulated with 2 or 20 ng/mL of recombinant human GDF15 (rhGDF15) for another 24 hours. Western blots of total protein lysates are shown for total IGF-1R with actin as loading control. Experiments were repeated 3 times; representative blots are shown. Quantification (shown beneath each band) was normalized to actin and performed using Odyssey Li-Cor imaging software. **(C)** JIMT-1 cells were pretreated for 24 hours with control IgG or 0.25 μg/ml alpha IR3 (aIR3) in serum-free media, and then stimulated with 2 or 20 ng/mL of recombinant human GDF15 (rhGDF15) for another 24 hours. Real-time PCR was performed for SNAIL, SLUG, MMP2, and MMP9. Values reflect average fold in transcript normalized to internal control RPLPO. Error bars represent standard deviation between triplicate samples; experiments were repeated 3 times. **(D)** JIMT-1 cells were pretreated for 24 hours with control IgG or 0.25 μg/ml alpha IR3 (aIR3) in serum-free media, and then seeded in Matrigel-coated Boyden chambers in serum-free media with 10% FBS in the lower chamber as chemoattractant. Drug treatment was continued (in indicated samples), and 2 or 20 ng/mL of recombinant human GDF15 (rhGDF15) was added to the chambers of treatment groups where indicated. After 24 hours of invasion, photos were taken at 20× magnification; representative photos are shown. The number of invaded cells is shown per group; error bars represent standard deviation between triplicate samples, ^*^p<0.05.

**Figure 8:**
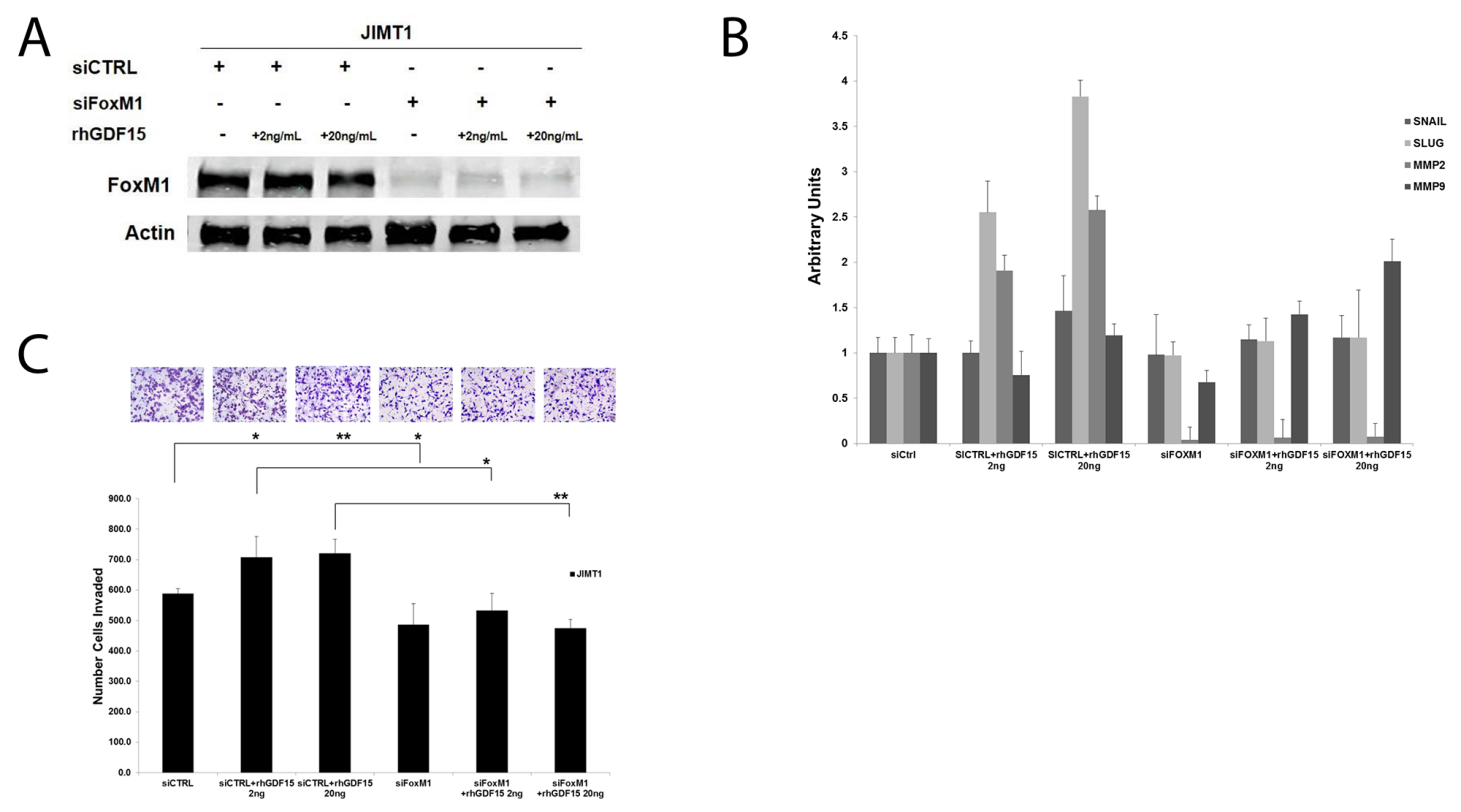
FoxM1 mediates GDF15-simulated EMT and invasion **(A)** JIMT-1 cells were transfected with 100 nM control siRNA (siCtrl) or FoxM1 siRNA (siFoxM1) for 24 hours, and then stimulated with 2 or 20 ng/mL recombinant human GDF15 (rhGDF15) for another 24 hours. Western blots of total protein lysates are shown for total FoxM1 with actin as loading control. **(B)** JIMT-1 cells were transfected with 100 nM control siRNA (siCtrl) or FoxM1 siRNA (siFoxM1) for 24 hours, and then stimulated with 2 or 20 ng/mL recombinant human GDF15 (rhGDF15) for another 24 hours. Real-time PCR was performed for Snail, Slug, MMP2, and MMP9. Values reflect average fold in transcript normalized to internal control RPLPO. Error bars represent standard deviation between triplicate samples; experiments were repeated 3 times. **(C)** JIMT-1 cells were transfected with 100 nM control siRNA (siCtrl) or FoxM1 siRNA (siFoxM1) for 24 hours, and then plated in serum-free media in Matrigel-coated Boyden chambers. Recombinant human GDF15 (rhGDF15; 2 or 20 ng/mL) was added to lower chambers where indicated. After 24 hours, cells were fixed and stained; representative photos of invading cells are shown at 20× magnification. The total number of invading cells was counted in 10 random fields; the average number of invading cells is shown for triplicate cultures per cell line, ^**^p<0.005, ^*^p<0.05.

Similar to stable GDF15-overexpressing BT474 clones, HER2-positive JIMT1 cells stimulated with 2 ng/mL or 20 ng/mL rhGDF15 exhibited a 1.5- to 2-fold increase in IGF-1R phosphorylation and expression (Figure 7A). Despite blocking rhGDF15-mediated induction of IGF-1R (Figure 7B), the IGF-1R antibody alpha IR3 only partially rescued the increased expression of mesenchymal markers (Figure 7C) and invasion (Figure 7D). Knockdown of FoxM1 (Figure 8A) rescued EMT (Figure 8B) and invasiveness (Figure 8C) of GDF15-overexpressing breast cancer cells.

### Knockdown of GDF15 inhibits breast cancer invasion

**Figure 9:**
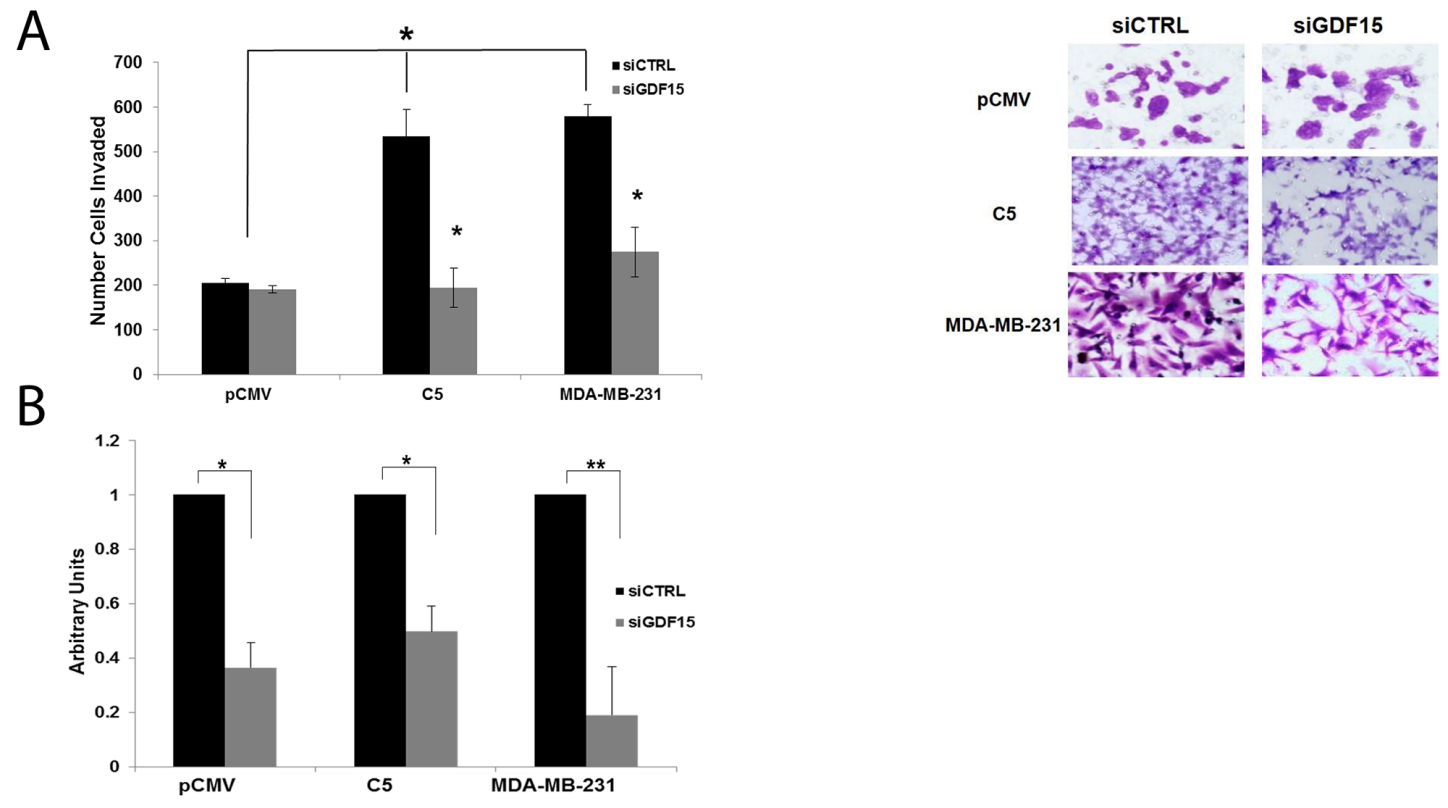
GDF15 knockdown inhibits invasion of breast cancer cells **(A)** BT474 stable empty vector control clone (pCMV), BT474 GDF15 stable clone 5 (C5), and MDA-MB-231 cells were transfected with 100 nM siRNA control (siCtrl) or GDF15 siRNA (siGDF15). After 24 hours, transfected cells were plated in serum-free media in Matrigel-coated Boyden chambers with 10% FBS in the wells as a chemoattractant. After 24 hours, cells were fixed and stained. Representative photos of invading cells are shown at 20× magnification. The total number of invading cells was counted in 10 random fields; the average number of invading cells is shown for triplicate cultures per cell line, ^*^p<0.05. **(B)** BT474 stable empty vector control clone (pCMV), BT474 GDF15 stable clone 5 (C5), and MDA-MB-231 cells were transfected with 100 nM siRNA control (siCtrl) or GDF15 siRNA (siGDF15) for 48 hours. Real-time PCR was performed to confirm GDF15 knockdown. Values reflect the fold change in transcript normalized to RPLPO housekeeping gene. Error bars represent standard deviation between triplicate samples; experiments were repeated twice; ^**^p<0.005, ^*^p<0.05.

Based on data indicating that GDF15 mediates EMT and invasion, we hypothesized that knockdown of GDF15 expression would block invasiveness of breast cancers that overexpress GDF15. Using siRNA, GDF15 was transiently knocked down in HER2-positive BT474 empty vector pCMV control, BT474 GDF15-overexpressing stable clone, and triple-negative MDA-MB-231 breast cancer cells, which have endogenous overexpression of GDF15 (Figure 9B). Knockdown of GDF15 significantly inhibited breast cancer invasion through basement membrane matrix (Figure 9A). These results establish a functional role for GDF15 in breast cancer invasion, and support further investigation into GDF15-targeting as a potential therapeutic strategy.

## DISCUSSION

**Figure 10:**
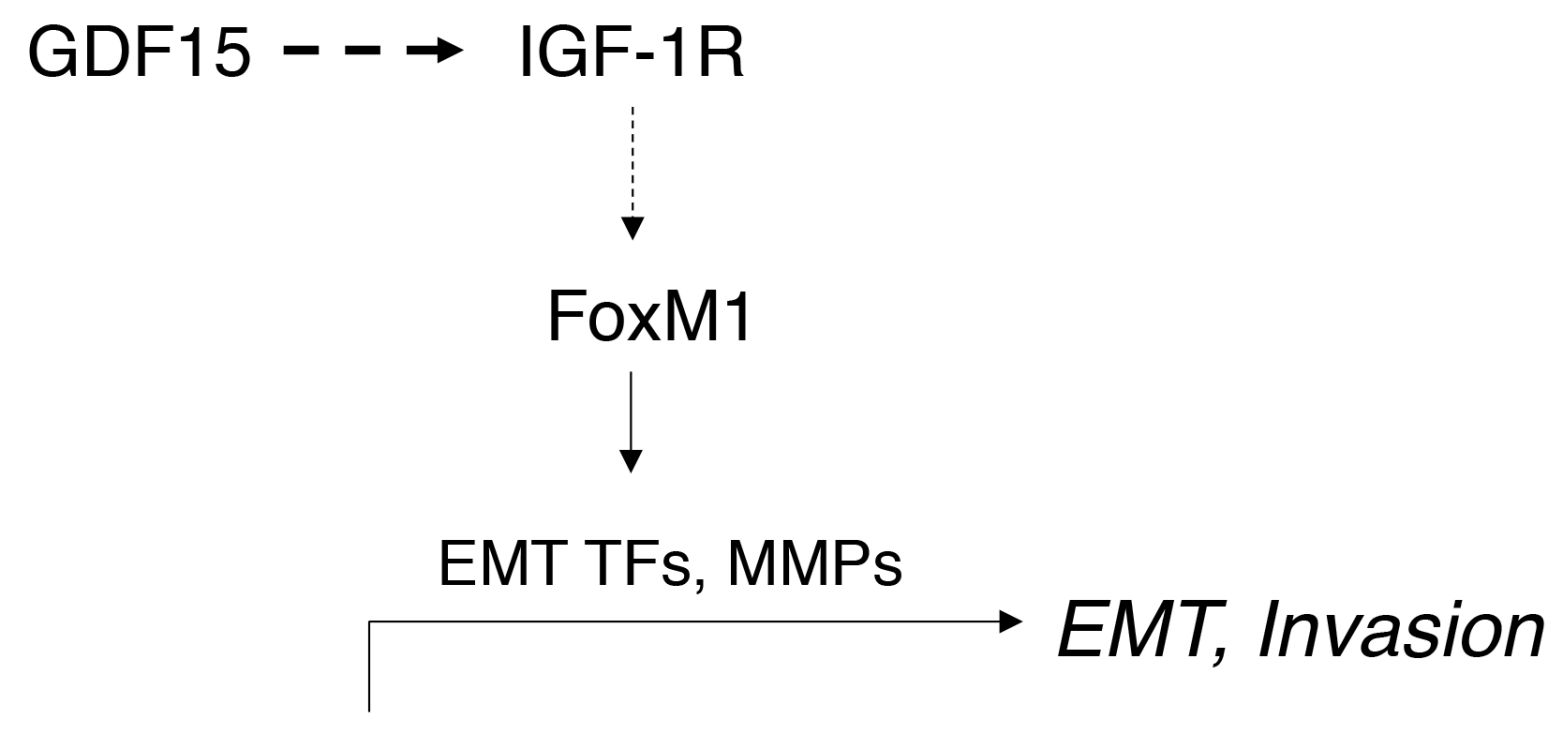
Schematic of model GDF15 activates IGF-1R signaling and induces expression of FoxM1, which upregulates expression of EMT transcription factors (TFs), including Snail and Slug, and induces expression of matrix metalloproteinases (MMPs), stimulating EMT and breast cancer cell invasion.

In the current study, we provide mechanistic insights into the signaling pathways driving GDF15-mediated EMT and invasion in breast cancer. GDF15 overexpression activated IGF-1R signaling with subsequent upregulation of FoxM1 and target MMPs, increased expression of EMT mediators, and increased cellular invasion (Figure 10). These data suggest that the inflammatory cytokine GDF15 contributes to breast cancer progression in part by activating signaling pathways that control EMT and cellular invasion.

IGF-1R activation and overexpression promote EMT in many tumor types. In breast cancer, IGF-1R signaling induces EMT, migration, and invasion in HER2-positive and triple-negative breast cancer [22, 24]. Acquisition of a mesenchymal phenotype has been reported to confer stem cell-like and migratory characteristics, resulting in recurrent metastases and drug resistance in multiple tumor models [25, 26]. Consistent with the GDF15-driven EMT we observed in our studies, GDF15 was recently shown to maintain stem cell characteristics in breast cancer cell lines [27]. The mechanisms through which IGF-1R promotes a switch to a mesenchymal phenotype are not fully understood. However, IGF-1R activation is known to stimulate MEK-Erk1/2 and PI3K-Akt signaling, which in turn regulate EMT transcription factors. We previously demonstrated that GDF15 induces PI3K and Erk1/2 phosphorylation in HER2-overexpressing breast cancer cells, in association with drug resistance [27]. Further, MEK inhibition blocked the ability of GDF15 to induce tumor sphere formation [16], indicating that MEK activation downstream of IGF-1R activation may contribute to GDF15-stimulated EMT. These data provide rationale for evaluating IGF-1R and MEK inhibition as a potential therapeutic strategy in mesenchymal GDF15-positive breast cancers.

In addition to regulating expression of EMT markers, IGF-1R inhibition blocked invasion of GDF15-overexpressing cells with reduced FoxM1 expression. We previously reported that FoxM1 is a critical mediator of IGF-1R-stimulated invasion in breast cancer cells [22]. The current study further supports FoxM1 as a mediator of IGF-1R-mediated invasion. FoxM1 knockdown reduced invasion, expression of mesenchymal transcription factors, and upregulation of MMP2 and MMP9 in BT474-derived GDF15 stable clones and JIMT1 cells. These data demonstrate that GDF15-IGF-1R-FoxM1 signaling stimulates breast cancer invasion, with IGF-1R and FoxM1 representing potential downstream targets for inhibiting GDF15-mediated invasion.

Additional mechanisms are likely to contribute to GDF15-mediated EMT and invasion. Some studies suggest that GDF15 induces phosphorylation of TGF-β signaling effectors Smad2 and Smad3 [14, 17], which are recognized mediators of EMT [28]. Recent studies identified the GDF15 receptor as GFRAL, a distant relative of TGF-β family members [11–13]. Thus, future evaluation of the role of GFRAL and associated signaling pathways in EMT and invasion of GDF15-overexpressing breast cancer cells is warranted. GDF15 also activates focal adhesion kinase (FAK) signaling, driving prostate cancer metastasis [29]. We previously reported that IGF-1R regulates FAK signaling and EMT in triple-negative breast cancer. Further, we and others previously have shown that GDF15 activates HER2 kinase activity [14, 24, 30], resulting in acquired and intrinsic resistance to HER2-targeted antibody therapy [27]. These results are consistent with previous reports that IGF-1R/HER2 cross-signaling causes trastuzumab resistance, and suggest that GDF15 overexpression may represent one mechanism driving activation of the HER2 and IGF-1R receptor kinase signaling network and drug resistance.

Although increased expression of GDF15 is associated with advanced disease in patients with multiple types of malignancies, limited data are available regarding the expression levels of GDF15 in breast cancer. Welsh et al. [10] examined changes in gene expression of secreted proteins in the sera of cancer patients representing 10 different tumor types. Serum levels of GDF15 were elevated in patients with metastatic colorectal (8/8), prostate (8/9), and breast (6/10) cancers when compared with sera from normal controls. Wollmann et al. [31] compared GDF15 transcript levels in 10 breast tumor samples matched with adjacent normal tissue controls, and found higher GDF15 expression in half of the tumor samples. Thus, according to these studies, which included a relatively small number of patient samples, GDF15 expression was elevated in 50% to 60% of serum or tumor tissue specimens from patients with breast cancer. Consistent with those studies, we demonstrate by IHC staining that GDF15 is expressed in 66% (397/605) of patients with breast cancer.

Sasahara et al. [16] recently reported that GDF15 expression was higher in breast cancer tissues compared with normal controls, with HER2-positive tumors demonstrating the highest expression levels of GDF15. We examined potential associations between GDF15 IHC staining and available clinical data, which included age, tumor size, tumor grade, disease involving >3 lymph nodes, ER and HER2 positivity, and overall survival. GDF15 expression was significantly associated with high tumor grade, and ER-negative or HER2-positive status. A limitation of our IHC study was that most patients in this cohort had low-grade tumors with either non-metastatic disease or fewer than 3 lymph node metastases. Thus, this cohort may represent a relatively low-risk population or subgroup with primarily localized or locally invasive disease. Future analyses should examine cohorts that include a greater percentage of patients with lymph node-positive or metastatic disease to determine if GDF15 overexpression is associated with metastasis, recurrence, or reduced survival rates in patients with advanced-stage disease, as is reported in other tumor types [32, 33]. These studies should also evaluate potential subtype-specificity of GDF15 expression in HER2-positive and ER-negative breast cancer, based on results presented here, and associations between GDF15 expression and treatment response or survival.

In summary, while GDF15 exhibits pleiotropic effects in cancer cells [7], the majority of studies support a role for GDF15 in disease progression, with overexpression linked to EMT, invasion and metastasis. Our studies are the first to report activation of IGF-1R-FoxM1 as a mechanism of invasion in GDF15-overexpressing breast cancers, providing rationale for preclinical evaluation of treatments that co-target IGF-1R and FoxM1. Further, based on the reduced invasiveness of HER2-positive and triple-negative breast cancers in response to GDF15 knockdown, future studies should evaluate GDF15 as a potential molecular target in breast cancer.

## MATERIALS AND METHODS

### Materials

The IGF-1R antibody alpha IR3 (Calbiochem; San Diego, CA) was provided at a stock concentration of 1 mg/mL. GDF15 small interfering RNA (siRNA; sc-39798) and control siRNA (sc-37007) (Santa Cruz Biotechnology; Dallas, TX) were resuspended in RNAse-free water. The IGF-1R antibody alpha IR3 (α-IR3; Calbiochem, San Diego, CA) was provided at a stock concentration of 1 mg/mL. Recombinant human GDF15 (rhGDF15; R&D Systems, 614 McKinley Place NE, Minneapolis, MN 55413) was dissolved in 4 mM HCl containing 0.1% BSA; the solvent (HCl-BSA) was used as vehicle control. The pan-MMP inhibitor GM6001 (Millipore; Temecula, Ca) was provided at a stock concentration of 1 mg/mL (2.5 mM) in DMSO.

### IHC

Breast tumor microarrays (TMAs) kindly provided by Dr. Fabrice Andre (Gustave Roussy Cancer Center, Villejuif, France) consisted of paraffin-embedded breast tumor tissues obtained from 605 patients diagnosed with breast cancer. Tumor tissues were spotted in triplicate per patient. Among 605 patients, 592 patients had recorded information for tumor size, grade, lymph node status, and ER status; thus, correlations were performed using data from these 592 patients. IHC staining was performed using a standard immunoperoxidase procedure as previously described [27]. Tissues were deparaffinized by heating at 60°C, passaging through xylene and alcohol grades, and ultimately water. Antigen retrieval was performed by boiling in 10 mM citrate buffer, pH 6.0 for 10 minutes (min), and cooling in the same buffer for 30 min. Endogenous peroxidase was quenched by incubating with 0.3% H_2_O_2_ in methanol for 15 min. After washing with water and PBS/TBS, TMA slides were incubated in 10% swine serum (Dako North America, Inc., 6392 Via Real, Carpinteria, CA 93013) for 1 hour (h) to block nonspecific background staining. TMAs were then stained with rabbit polyclonal anti-human GDF15 (also known as MIC-1; antibody 3249; dilution 1:100; Cell Signaling Technology, Inc., 3 Trask Lane, Danvers, MA 01923) overnight at 4°C. Secondary antibody staining was then performed with biotinylated anti-rabbit antibody (Dako North America, Inc.), followed by visualization with 3,3-diaminobenzidine solution (DAB+ chromogen; Dako North America, Inc.) and hematoxylin as a counterstain. Slides were washed in water, dehydrated by passing through alcohol grades and xylene, and mounted with Permount (Fisher Scientific, 300 Industry Dr., Pittsburgh, PA 15275). GDF15 staining intensity was viewed under a light microscope, and scored in a blinded manner from 0 to 3 by a breast pathologist at Emory University, i.e., the pathologist had no knowledge of molecular or clinical characteristics of the tumor samples. Correlations between GDF15 score and clinical characteristics were determined by two-tailed Fisher’s exact test, with P<0.05 considered statistically significant.

### Cell culture

JIMT1 cells were purchased from DSMZ (Braunschweig, Germany); all other cell lines were purchased from American Type Culture Collection (Manassas, VA). MDA-MB-231, JIMT-1, and BT474 cells were maintained in Dulbecco’s Modified Eagle’s Medium (DMEM) with 4.5 g/L glucose, glutamine, and sodium pyruvate (Corning; Manassas, VA) with 10% FBS and 1% penicillin/streptomycin. All cells were cultured in humidified incubators at 37°C with 5% CO_2_.

#### Creation of stable GDF15-overexpressing clones

BT474 cells were transfected with 3 μg plasmid DNA (pCMV empty vector or pCMVmyc-GDF15, both from Origene, Rockville, MD) using Lipofectamine (Invitrogen, Carlsbad, CA) and DMSO shock. After 24–36 h, cells were maintained in 200 μg/mL of G418 to select successfully transfected cells. After approximately 2–3 weeks, multiple surviving clones were isolated and tested by real-time PCR for GDF15 expression.

#### Transfection

Cells were plated in antibiotic-free media at a concentration of 2×10^5^ cells/mL. The next day, cells were transfected using Lipofectamine 2000 (Invitrogen; Carlsbad, CA) with 100 nM GDF15 siRNA, 100 nM FOXM1 siRNA, or control siRNA (Santa Cruz Biotechnology) according to the manufacturer’s protocol. Media was changed after 6 h of transfection and replaced with complete media. Cells were harvested after 24 or 48 h.

#### Cell stimulation

Cells were plated and serum starved for at least 24 h prior to stimulation with vehicle control, 2 or 20 ng/mL of rhGDF15 for varying time points. Experiments were repeated at least twice.

#### Spheroid migration assays

Cells were seeded (5×10^4^) in 1% agar-coated 96-well plates and cultured for 48 h in a humidified atmosphere containing 5% CO_2_ at 37°C. Intact tumor spheroids were carefully transferred to a 96-well plate and cultured in complete media for 24 h. Spheroids and migrated cells were fixed with 100% methanol, stained with 0.05% crystal violet, and observed using a normal light microscope (20×) and Olympus DP-30BW digital camera. Eight replicates were included per group, and experiments were repeated three times.

#### Invasion assays

Cells were plated in serum-free media in BD BioCoat Matrigel Invasion Chambers (BD Biosciences; Franklin Lakes, NJ) (1×10^5^ cells/mL) with 0.75 mL chemoattractant (culture media containing 10% FBS) in each well. Depending on the experiment, cells were pre-treated with control mouse IgG or alpha IR3 (0.25 μg/mL) added directly to the chamber, or transfected with control siRNA or GDF15 siRNA overnight prior to placing cells in invasion chambers. Non-invading cells were removed from the interior surface of the membrane by scrubbing gently with dry cotton tipped swab. Each insert was then transferred into 100% methanol for 10 minutes followed by crystal violet staining for 20 minutes. Membranes were washed in water, allowed to air dry completely before being separated from the chamber, and then mounted on slides with permanent mounting medium Permount (Fisher Scientific). Multiple photographs of each sample were taken at 20× magnification, with triplicates per treatment group. The number of cells was counted in each field, and the sum of cells in all fields was calculated for each sample. Triplicate cultures were included per group, and experiments were repeated at least twice.

### Cell cycle analysis

Cells were harvested, washed twice with DPBS + 10% FBS, fixed in ice-cold 80% ethanol, and stored at -20°C for at least 24 hours. Fixed cells were incubated in 50 μL propidium iodide buffer (20 μg/mL PI (Sigma), 0.1% Triton-X 100, 200 μg/mL RNaseA (Promega) in DPBS) for 30 minutes in the dark. Cells were resuspended in 400 μL DPBS. Samples were analyzed using a BD FACS Canto II cytometer (BD Biosciences; San Jose, CA) and BD FACS Diva software. Triplicates were included per group, and experiments were repeated at least twice.

### Western blotting

Cells were lysed in RIPA buffer (Cell Signaling; Danvers, MA) supplemented with protease and phosphatase inhibitors (Sigma-Aldrich). Total protein extracts were run on SDS-PAGE and blotted onto nitrocellulose. Blots were probed overnight using the following antibodies from Cell Signaling: rabbit anti-phospho-IGF-1 receptor β (Tyr1131) (#3021, 1:200); rabbit anti-IGF-1 receptor β (#3018, 1:250); and rabbit-anti-N-Cadherin (#4061, 1:1000). Rabbit anti-GDF15 (#8479 1:200) was purchased from AbCam (Cambridge, MA). Mouse anti-E-Cadherin (#610181, 1:1000) was purchased from BD Biosciences (San Jose, CA). Mouse anti-vimentin (Sigma-Aldrich; V6630) was used at 1:1000. Mouse anti-β-actin monoclonal AC-15 (Sigma-Aldrich) was used at 1:10,000 as a loading control. All primary antibodies were diluted in 5% BSA/TBS-T. Goat anti-mouse secondary IRDye 800 antibody (#926-32210, 1:10,000) was purchased from Li-Cor Biosciences (Lincoln, NE). Goat anti-rabbit Alexa Fluor 680 secondary antibody (#1027681, 1:10,000) was purchased from Invitrogen (Grand Island, NY). Protein bands were detected using the Odyssey Imaging System (Li-Cor Biosciences, Lincoln, NE). Blots were repeated at least three times.

### Quantitative RT-PCR

Total RNA was extracted using the RNeasy purification kit (Qiagen; Valencia, CA) and treated with DNase (Invitrogen; Carlsbad, CA). Total RNA was used to prepare cDNA using random primers and the Superscript III first strand synthesis kit (Invitrogen; Carlsbad, CA). Relative levels of mRNA were determined by real-time quantitative PCR using an Applied Biosystems cycler and the TaqMan Universal PCR master mix (4304437; Applied Biosystems; Carlsbad, CA). Primers for RPLPO (Hs99999902_M1), FOXM1 (Hs01073586_M1), GDF15 (Hs00171132_m1), MMP2 (Hs01548733_m1), MMP9 (Hs00234579_m1), E-Cadherin (Hs01023894_m1), SNAIL (Hs00195591_m1), SLUG (Hs00950344_m1), and Zeb-1 (Hs00232783_m1) were obtained from Applied Biosystems (Taq-Man Gene Expression Assays; Carlsbad, CA). After amplification, data were normalized to RPLPO levels and analyzed by delta Ct method. Triplicates were included per group, and experiments were repeated at least twice.

### Statistics

P-values were determined for experimental versus control treatments by two-tailed student’s t-test, ^*^p<0.05, ^**^p<0.005. IHC correlations were determined by two-tailed Fisher’s exact test.
