# Correction: Growth differentiation factor 15 mediates epithelial mesenchymal transition and invasion of breast cancers through IGF-1R-FoxM1 signaling

**DOI:** 10.18632/oncotarget.27779

**Published:** 2020-11-03

**Authors:** Bridgette F. Peake, Siobhan M. Eze, Lily Yang, Robert C. Castellino, Rita Nahta

**Affiliations:** ^1^ Molecular & Systems Pharmacology PhD Program, Graduate Division of Biological and Biomedical Sciences, Emory University, Atlanta, GA, USA; ^2^ Department of Pharmacology, School of Medicine, Emory University, Atlanta, GA, USA; ^3^ Department of Pediatrics, School of Medicine, Emory University, Aflac Cancer & Blood Disorders Center, Children’s Healthcare of Atlanta, Atlanta, GA, USA; ^4^ Department of Hematology & Medical Oncology, School of Medicine, Emory University, Atlanta, GA, USA; ^5^ Department of Surgery, School of Medicine, Emory University, Atlanta, GA, USA; ^6^ Winship Cancer Institute, Emory University, Atlanta, GA, USA

**This article has been corrected:** Due to mistakes during the assembly of Figure 6, the cell images at the top of Figures 6A and 4C are accidental duplicates. Figure 4C is correct as presented. The corrected Figure 6A, using the proper images obtained from the original data, is shown below. The authors declare that these corrections do not change the results or conclusions of this paper.

Original article: Oncotarget. 2017; 8:94393–94406. 94393-94406. https://doi.org/10.18632/oncotarget.21765

**Figure 6:**
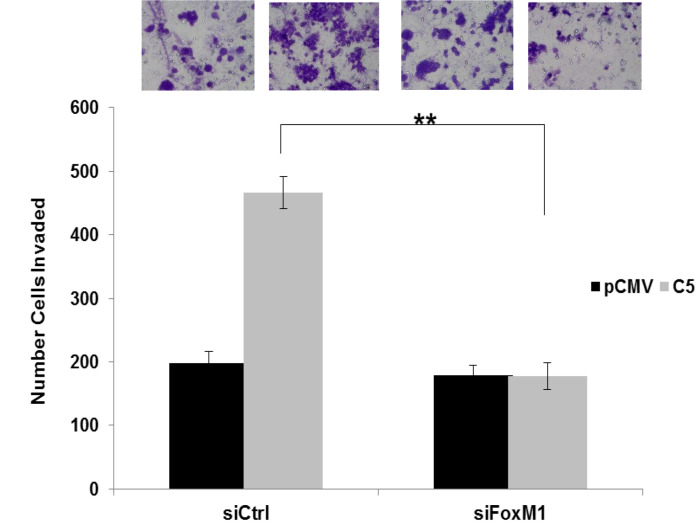
FoxM1 promotes invasion and upregulation of MMP2 and MMP9 in GDF15-overexpressing breast cancer cells. (**A**) BT474 stable empty vector control clone (pCMV) and GDF15 stable clone 5 (C5) were transfected with 100 nM control siRNA (siCtrl) or FoxM1 siRNA (siFoxM1) for 48 hours, and then plated in serum-free media in Matrigel-coated Boyden chambers. After 24 hours, cells were fixed and stained. Representative photos of invading cells are shown at 20× magnification. The total number of invading cells was counted in 10 random fields; the average number of invading cells is shown for triplicate cultures per cell line; student’s *t*-test, ^**^ *p* < 0.005.

